# Prognostic value of controlling nutritional status on clinical and survival outcomes in cancer patients treated with immunotherapy

**DOI:** 10.1038/s41598-023-45096-1

**Published:** 2023-10-18

**Authors:** Jiacheng Zhang, Man Li, Lilong Zhang, Tianrui Kuang, Jia Yu, Weixing Wang

**Affiliations:** 1 Department of Hepatobiliary Surgery, Renmin Hospital of Wuhan University, Wuhan, Hubei Province People’s Republic of China; 2 Central Laboratory, Renmin Hospital of Wuhan University, Wuhan, Hubei Province People’s Republic of China; 3 Department of General Surgery, Renmin Hospital of Wuhan University, Wuhan, Hubei Province People’s Republic of China

**Keywords:** Risk factors, Prognostic markers, Immunotherapy, Cancer immunotherapy, Tumour biomarkers

## Abstract

Cancer is a leading cause of death globally. Immunotherapy has shown promise in treating various types of cancer, but its effectiveness varies among patients. The Controlling Nutritional Status (CONUT) score has been linked to the prognosis of different cancers. However, its predictive value for immunotherapy outcomes is not well understood. Our research represents the pioneering meta-study to examine the prognostic value of the CONUT score on cancer patients treated with an immune checkpoint inhibitor (ICI). A comprehensive literature search was conducted using various databases including PubMed, the Cochrane Library, EMBASE, and Google Scholar. The study was conducted until July 28, 2023. This analysis encompassed a comprehensive evaluation of various clinical outcomes, namely overall survival (OS), progression-free survival (PFS), objective response rate (ORR), and disease control rate (DCR). 663 patients from 8 studies were included in this study. It showed that cancer patients with high CONUT score had poorer OS (HR: 1.94, 95% CI, 1.52–2.47, *p* < 0.001) and PFS (HR: 2.22, 95% CI, 1.48–3.31, *p* < 0.001), as well as worse ORR (OR: 0.46, 95% CI, 0.25–0.85, *p* = 0.013) and DCR (HR: 0.29, 95% CI, 0.14–0.59, *p* = 0.001). The CONUT score can predict the prognosis of tumor patients treated with ICIs.

## Introduction

One of the leading causes of death in the globe is cancer, only second to cardiovascular disease^1,2^. In just one year in 2020, 9.3 million new cancer cases were found around the globe, with 10 million deaths caused by cancer^2^. According to the GLOBOCAN statistics, the worldwide cancer burden is growing. Each year, 29.5 million new cancer diagnoses are projected by 2040, with 16.5 million cancer-related deaths (https://gco.iarc.fr/tomorrow), revealing a major burden on society and the economy. Great progress has been made in the treatment of cancer. Current therapeutic modalities for cancer management encompass a range of interventions, namely surgical resection, radiation therapy, chemotherapy, hormone therapy, Chinese medicine therapy, and immunotherapy^3^. Immune checkpoint inhibitors (ICI) therapy is a type of immunotherapy that targets drugs that inhibit PD1(Programmed Death-1), PD-L1(Programmed Death Ligand-1), and CTLA-4(Cytotoxic T Lymphocyte-associated Antigen-4) and has demonstrated encouraging outcomes in the treatment of a variety of tumors^4,5^, including non-small-cell lung cancer(NSCLC)^6^, renal cell carcinoma (RCC)^7^, hepatocellular carcinoma (HCC)^8^, and melanoma^9^. However, various individuals' immune responses are variable, resulting in low immunological effectiveness in certain patients^10^. As a result, the development of robust biomarkers with high predictive value for assessing the prognosis of cancer patients following immunotherapy is of paramount importance, allowing for individualized and accurate immunotherapy.

It is generally understood that the patient's immune nutritional condition is critical in cancer therapy^11^. Systemic inflammation and malnutrition are important prognostic indicators for malignant cancers^12,13^. Multiple nutritional assessment systems, including Nutritional Risk Screening (NRS), albumin (ALB), and prognostic nutritional index (PNI), have been shown to predict the prognosis of tumor patients^14,15^. The Controlling Nutritional Status (CONUT) score, is an innovative and straightforward clinical nutritional index, consisting of three blood measures: lymphocytes, albumin, and total cholesterol^16^. Ignacio et al. first introduced the CONUT score as a standard evaluation technique to evaluate the nutritional status of hospitalized patients ^16^ (The scoring criteria can be seen in Table 1). The CONUT scores are closely linked to the prognosis of diverse forms of malignancies, such as colorectal cancer, esophageal squamous cell carcinoma (ESCC), glioblastoma, gynecological cancer, pancreatic ductal adenocarcinoma, and gastric cancer (GC)^17–22^. Recently, CONUT has also been applied in predicting tumor immunotherapy. CONUT score was an independent predictor of the efficacy of treatment and OS in NSCLC^23^. However, no independent predictive effect was observed in gastric cancer^24^. To date, the prognostic value of novel inflammatory biomarker CONUT for ICIs is unknown in most tumor types, and no meta-analysis has been performed.

**Table 1:** The scoring system of CONUT score.

Parameter	Ranger of values and scores per parameter			
Serum albumin (g/dL)	≥ 3.50	3.00–3.49	2.50–2.99	< 2.5
Score	0	2	4	6
Lymphocyte count (/ul)	≥ 1600	1200–1599	800–1199	< 800
Score	0	1	2	3
Total cholesterol (mg/dL)	≥ 180	140–179	100–139	< 100
Score	0	1	2	3
CONUT score	0–1	2–4	5–8	9–12
Degree of malnutrition	None	Light	Moderate	Severe

This research conducted a systematic evaluation of the predictive effect of CONUT in the treatment of cancers by ICIs.

## Methods

### Literature search strategies

This analysis was carried out with the PRISMA declaration^25^. On July 28, 2023, a thorough literature search was performed using several databases, such as PubMed, EMBASE, the Cochrane Library, and Google Scholar. Our search strategy was from the date of search construction to the date of search completion. Relevant papers were retrieved using several search phrases, encompassing MeSH terms and keywords, for example, “Immune Checkpoint Inhibitors [MeSH]”, “Checkpoint Blockade, Immune”, “Blockade, PD-1-PD-L1”, “CTLA-4 Inhibitors”, “Tislelizumab”, “Toripalimab”, “Envafolimab”, “Camrelizumab”, “Sintilimab”, “Nivolumab”, “Controlling Nutritional Status”, “CONUT”. Specific search strategies are available in the supplementary file. The search parameters have been restricted to the field of English literature only. A comprehensive elucidation of the search methodologies is presented in Table 2. In addition, a comprehensive search was conducted for grey literature utilizing the Google Scholar database. Grey literature was a Google preprint that failed to be published due to negative results. Furthermore, all qualified studies' reference lists underwent a careful manual screening.

**Table 2:** Main characteristics of the studies included.

References	Study design	Study period	Study region	ICI treatment	Cancer Type	Sample size	Age (years)	Gender (male/female)	Outcome	NOS
Chang et al.^26^	R	01/2017–10/2020	China	Camrelizumab, Sintilimab, Toripalimab	EC	69	60 (44–78)^a^	67/2	OS (M), PFS (M), ORR (U), DCR (U)	7
Chen et al.^24^	R	08/2016–12/2020	China	ICIs	GC	89	–	–	OS (U), PFS (U)	7
Chen et al.^27^	S	06/2019–09/2020	China	Sintilimab	HCC	20	56 (41–70)^a^	18/2	PFS (U)	6
Ohba et al.^23^	R	02/2017–01/2018	Japan	Pembrolizumab	NSCLC	32	65 (44–85)^a^	29/3	OS (M), PFS (M), ORR (U), DCR (U)	7
Sakai et al.^28^	R	04/2017–06/2022	Japan	Nivolumab, Pembrolizumab	HNSCC	102	70 (47–87)^a^	93/9	OS (U), PFS (U), ORR (U), DCR (U)	8
Takemura et al.^29^	R	2016–2019	Japan	Nivolumab	RCC	60	68 (60–76)^b^	45/15	OS (U), PFS (U)	7
Zhang et al.^30^	R	2019–2021	China	Camrelizumab, Toripalimab, Pembrolizumab	ESCC	243	–	–	OS (U), PFS (U)	8
Zhao et al.^31^	R	01/2018–04/2021	China	Camrelizuma	ESCC	48	65^c^	32/16	OS (U), PFS (M), ORR (U)	7

^a^Medians (ranges).

^b^Medians (interquartile range).

^c^Medians.

*R* retrospective study, *S* single-arm study, *M* multivariate analysis, *U* univariate analysis, *ICIs* immune checkpoint inhibitors, *EC* esophageal cancer, *GC* gastric cancer, *HCC* hepatocellular carcinoma, *NSCLC* non-small cell lung cancer, *HNSCC* squamous cell carcinoma of head and neck, *RCC* renal cell carcinoma, *ESCC* esophageal squamous cell carcinoma, *OS* overall survival, *PFS* progression-free survival, *ORR* objective response rate, *DCR* disease control rate.

### Study selection criteria

In this research, we rigorously incorporated research that satisfied the subsequent standards: people with cancer and treated with ICIs and evaluated the CONUT score’s value of prediction. Moreover, the aforementioned articles have documented the occurrence of at least one of the subsequent outcomes: OS, PFS, ORR, and DCR. Several other types of articles, for instance, conference abstracts, and case reports, were not included in our analysis. In cases where there is patient overlap in the research, we prioritize studies that have the most thorough data and reliable methods^32^.

### Data extraction and quality evaluation

Two researchers manner-independently extracted the data. Any discrepancies were resolved through discussion until a consensus was reached. The collected data comprises the primary author's name, year of publication, article design, article period, treatment, cancer type, sample capacity, age, outcome, and so on. The Newcastle–Ottawa Scale (NOS) was conducted to evaluate the quality of the individual research^33^. Research with NOS values surpassing 6 were categorized as studies of superior quality.

### Statistical analysis

The terminal point of this meta-analysis was to predict medical outcomes for cancer patients after ICItreatment, covering OS, PFS, ORR, and DCR. The correlation between the CONUT and cancer outcome after ICI treatment was established by amalgamating the HR and the corresponding 95% CI for each included study. The heterogeneity among the studies was assessed utilizing Cochran's Q test and Higgins I^2^ statistics. As in previous studies, in cases where there was notable heterogeneity (I^2^ > 50% or *p* < 0.1), the combined analysis was assessed with the random-effects model (REM) as per the DerSimonian and Laird method. Alternatively, in the absence of any notable heterogeneity, the fixed-effects model (FEM) was employed with the Inverse Variance method^13^. To assess the origin of heterogeneity and dependability of the findings, we performed a sensitivity analysis. Assessment of publication bias was conducted utilizing various statistical methods, including the examination of Egger’s test^34^ and Begg’s test^35^. The results were shown with funnel plots and the calculation results were also shown in the supplementary file.

## Results

### Literature search process and results

**Figure 1:**
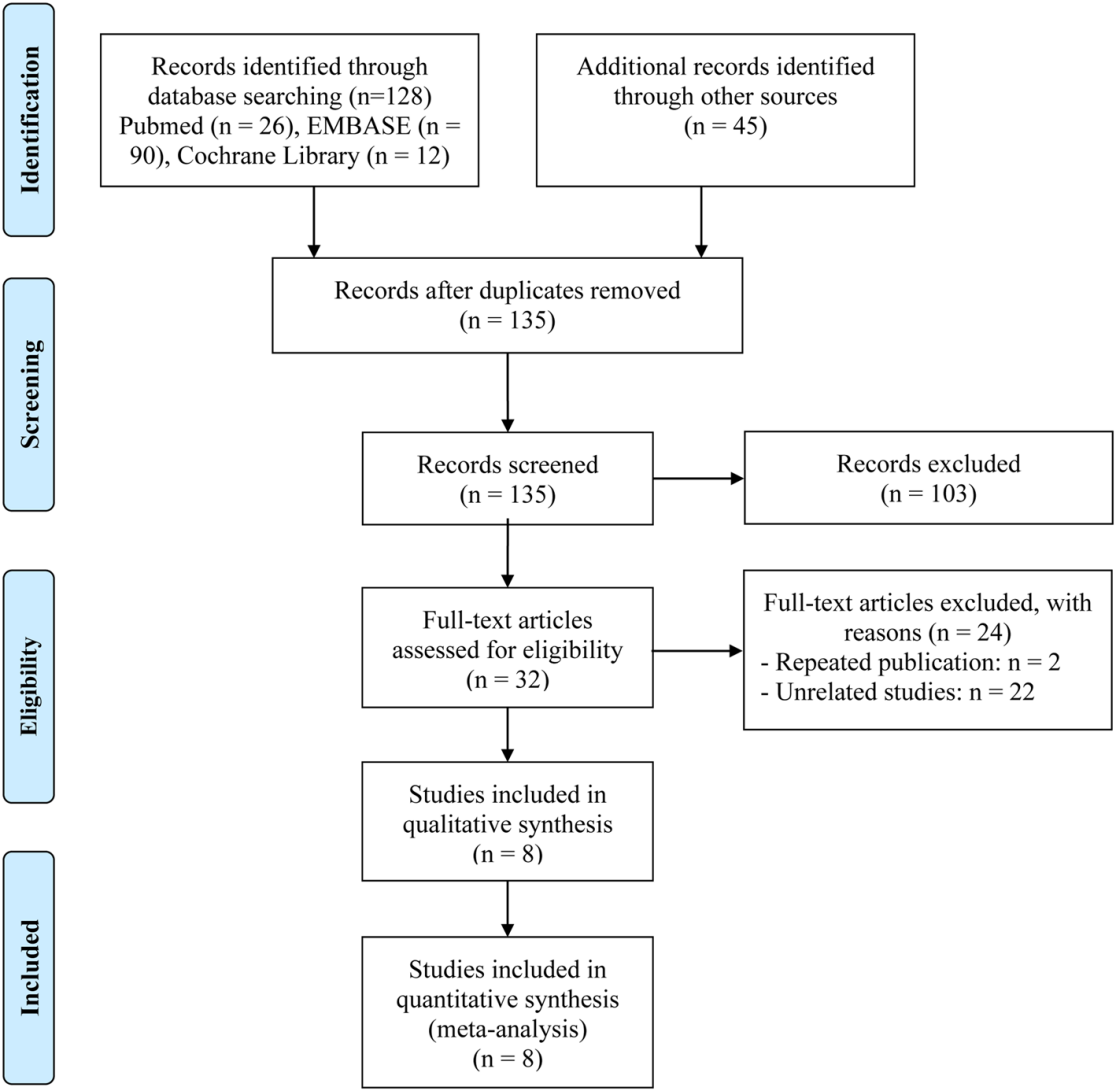
The flow diagram for identifying eligible studies.

PRISMA flow diagram of the article selection process was summarized in Fig. 1. Upon conducting an initial literature search by reviewing pertinent databases, a sum of 173 articles was determined. Following the elimination of 38 duplicate articles, 135 unique articles remained for subsequent evaluation. 103 articles were deemed unsuitable for further consideration based on an evaluation of their title and abstract within the literature. Upon thorough examination, a total of 8 articles with complete text, including 663 patients were ultimately incorporated into our meta-analysis^23,24, 26–31^.

### Characteristics of contained articles

The salient features of the incorporated articles are succinctly outlined in Table 2. Three of these investigations were conducted in Japan^23,28, 29^, and five in China^24,26, 27, 30, 31^. The range of NOS scores observed in the research encompassed values between 6 and 9, signifying that all included studies exhibited a high level of quality. All seven studies included in the analysis were retrospective^23,24, 26, 28–31^, while one was single-arm study^27^. Two studies were conducted in patients diagnosed with ESCC patients^30,31^; one study was in HCC patients^27^, one study was in esophageal cancer (EC) patients^26^, one study was in GC patients^24^, one study was in NSCLC patients^23^, one study was in squamous cell carcinoma of the head and neck (HNSCC) patients^28^, and one study was in RCC patients^29^. Furthermore, 7 studies reported CONUT’s role in prognosticating OS outcome^23,24, 26, 28–31^, all 8 studies provided the data of CONUT for PFS prognosis^23,24, 26–31^, 3 studies provided the data of CONUT for DCR prognosis^23,26, 28^, and 4 studies provided the data of CONUT for ORR prognosis^23,26, 28, 31^.

### Baseline CONUT levels and OS

**Figure 2:**
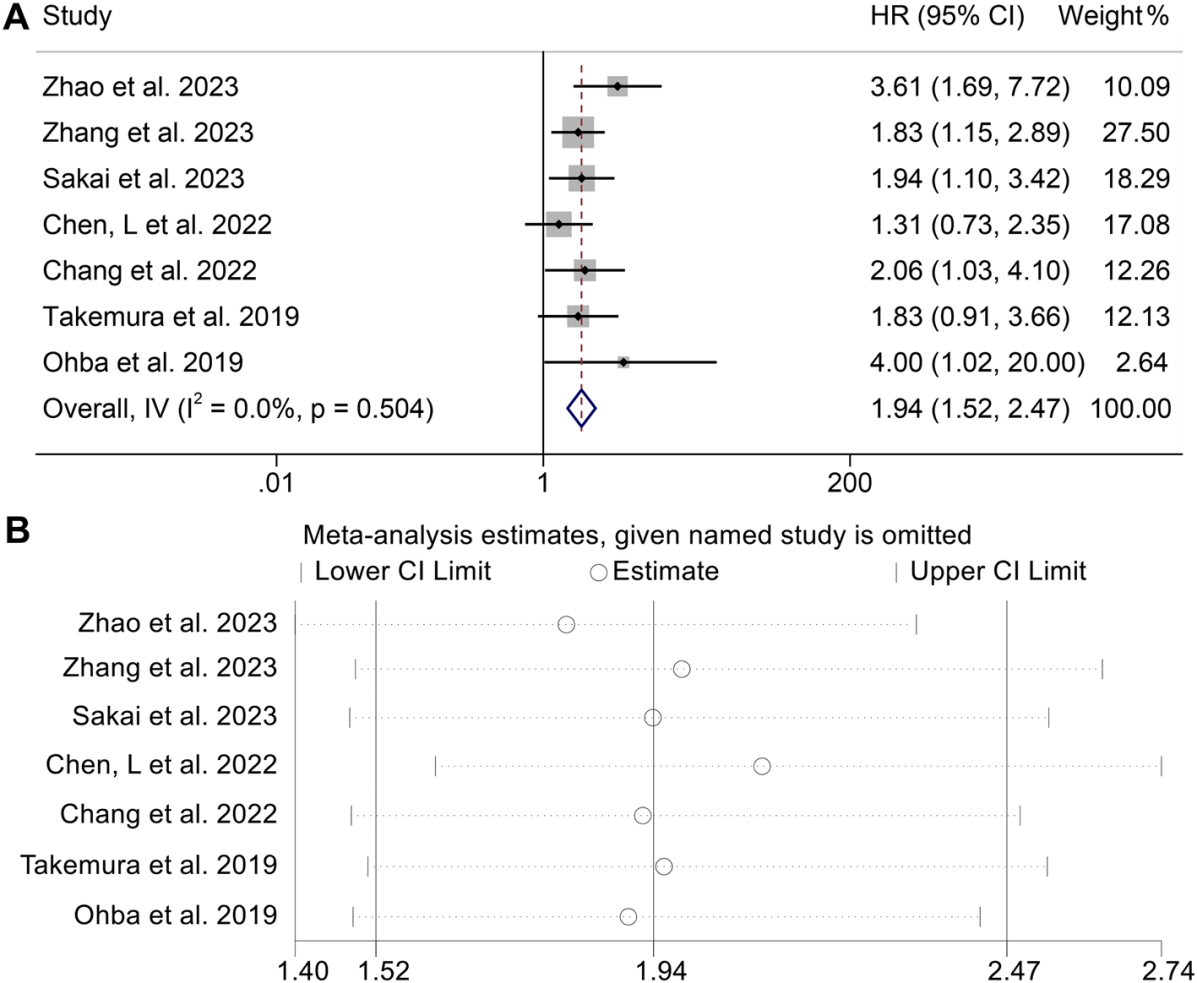
(**A**) Forest plots of the association between Controlling Nutritional Status (CONUT) and overall survival (OS). HR, hazard ratio; Cl, confidence interval. (**B**) Sensitivity analysis of the association between CONUT and OS.

**Figure 3:**
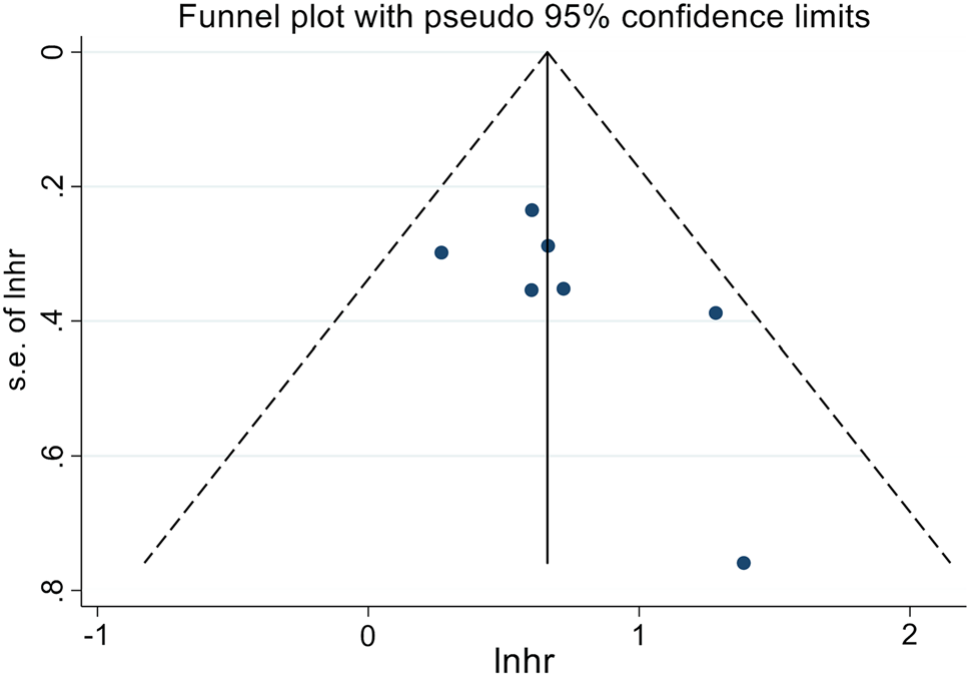
Funnel plot with pseudo 95% confidence limits. S.e. of: theta, the corresponding standard error.

In this study, our objective was to explore the potential correlation between CONUT levels and OS in cancer patients who were treated using ICIs. To achieve this, we conducted a comprehensive analysis of data obtained from seven independent studies, which included a total of 643 patients. The FEM was utilized as a result of the absence of significant heterogeneity (I2 = 0%, *p* = 0.504). As shown in Fig. 2A, the pooled HR was 1.94 (95% CI 1.52–2.47, *p* < 0.001) and indicated that a high CONUT score had poorer OS in cancer patients treated with immunotherapy. To assess the validity of the study's results, a sensitivity analysis was conducted. This involved examining the impact on the final results after eliminating each research from the analysis. Sensitivity analysis revealed that none of the studies significantly impacted the reported effect magnitude (Fig. 2B). When Zhao et al. was excluded, the estimated range of HR for OS changed to 1.81 (95%CI: 1.40–2.33), and when Chen et al. 2022 was excluded, the estimated range was 2.01 (95%CI: 1.61–2.74) (Fig. 2B). No publication biases in OS were observed in our study (Fig. 3, Egger's test: *p* = 0.133; Begg’s test: *p* = 0.149).

### Baseline CONUT levels and PFS

**Figure 4:**
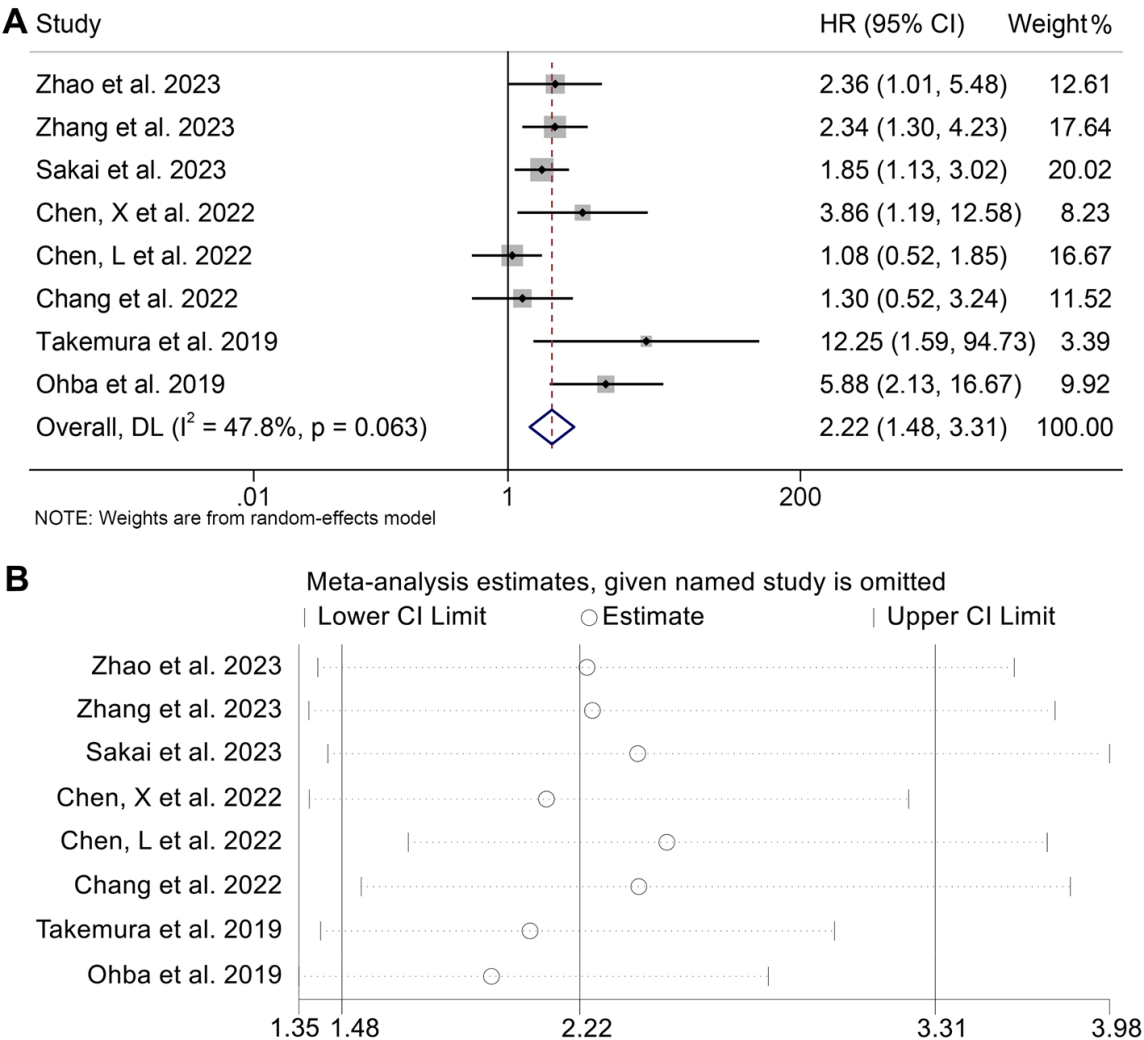
(**A**) Forest plots of the association between CONUT and progression-free survival (PFS). *HR*, harzard ratio; *Cl*, confidence interval (**B**) Sensitivity analysis of the association between CONUT and PFS.

**Figure 5:**
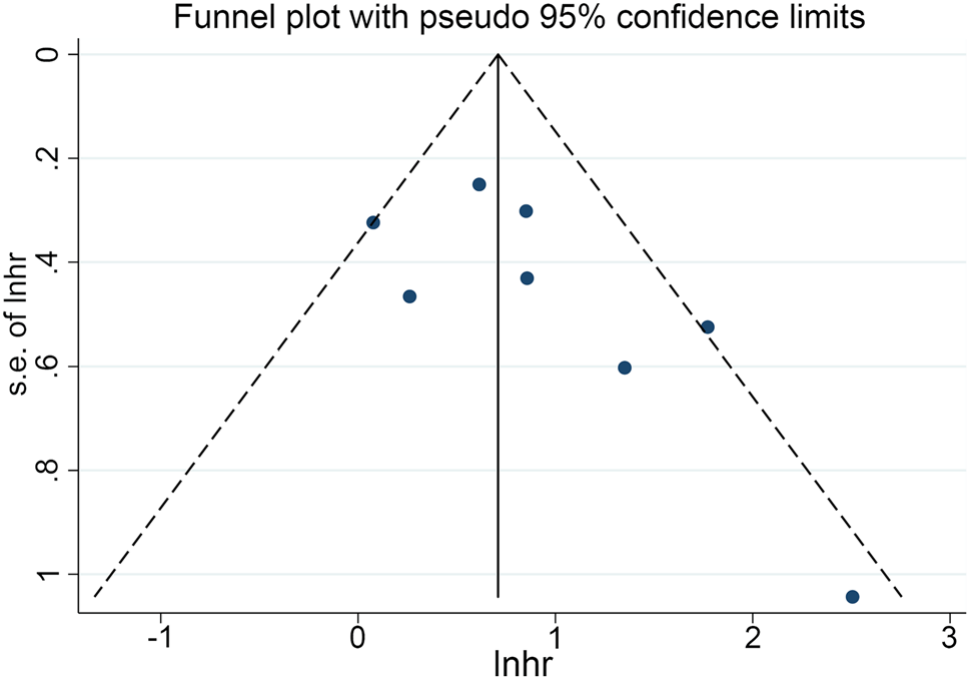
Funnel plot with pseudo 95% confidence limits in PFS.

Furthermore, the connection between CONUT score and PFS in ICI-treated cancer patients was explored by examining the data from all 8 studies with 663 patients. Because of the significant heterogeneity, the REM was selected for further analysis (I2 = 47.8%, *p* = 0.063). According to Fig. 4A, the merged HR was 2.22 (95% CI 1.48–3.31, *p* < 0.001), indicating that a high CONUT score had worse PFS in cancer patients treated with immunotherapy. As shown in Fig. 4B, the exclusion of any specific study didn’t have an impact on the overall findings about PFS. We also observed no publication biases in PFS (Fig. 5, Egger's test: *p* = 0.174; Begg’s test: *p* = 0.093).

### Baseline CONUT levels and ORR

**Figure 6:**
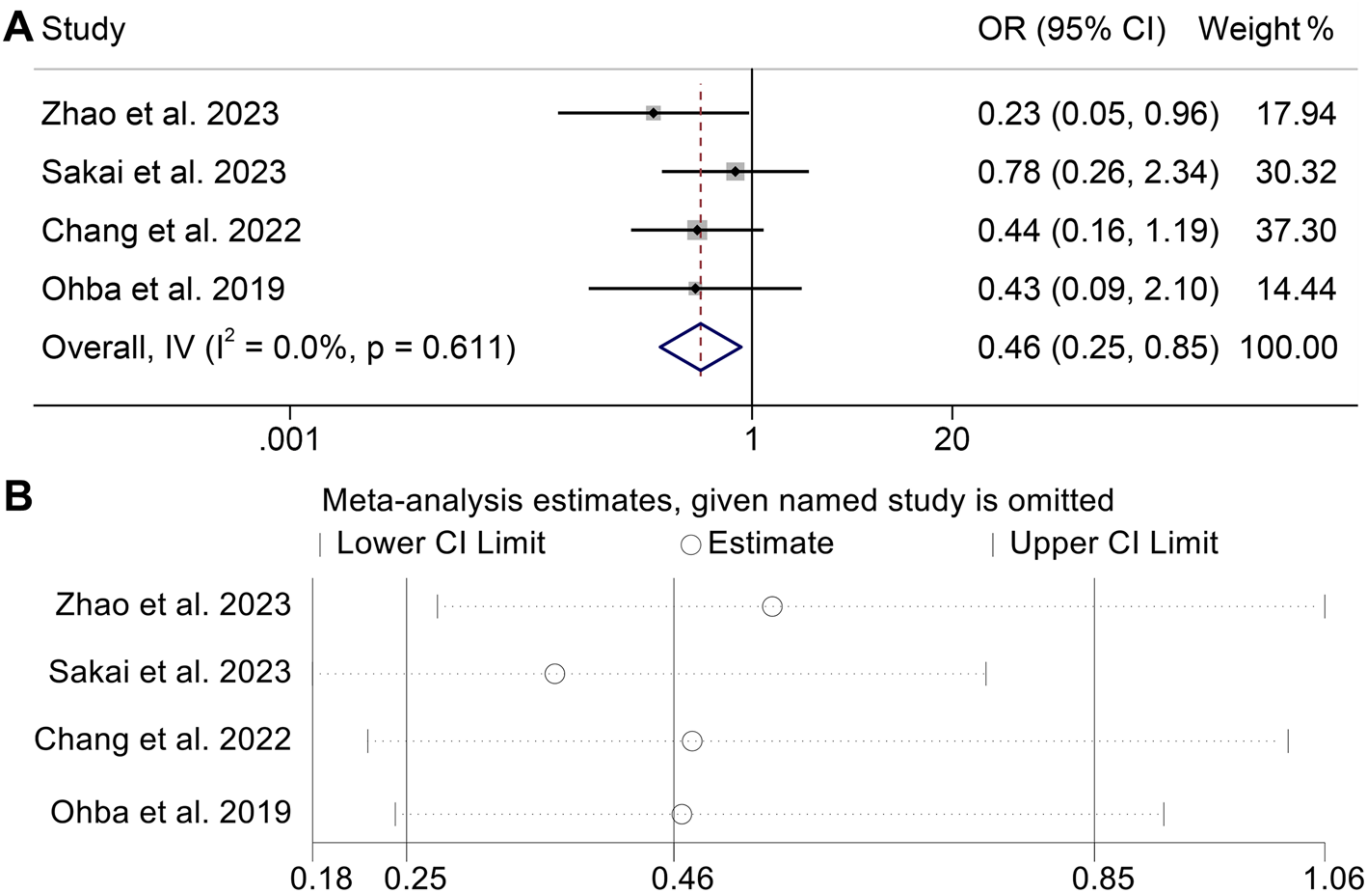
(**A**) Forest plots of the association between CONUT and objective response rate (ORR). *OR*, odds ratio; *Cl*, confidence interval (**B**) Sensitivity analysis of the association between CONUT and ORR.

We additionally examine the association between CONUT score and ORR in cancer patients undergoing treatment with ICIs, utilizing data obtained from four studies encompassing a cohort of 251 patients. Since the heterogeneity of the included studies was not significant, a fixed-effect model was selected for further analysis (Fig. 5, I2 = 0%, *p* = 0.611). Based on the findings presented in Fig. 6A, the OR was 0.46 (95% CI 0.25–0.85, *p* = 0.013), indicating that a high CONUT score had lower ORR in cancer patients treated with immunotherapy. As Fig. 6B demonstrated, the overall result of ORR was unaffected by the removal of any one research. Moreover, publication bias was not found in the ORR (Egger's test: *p* = 1; Begg’s test: *p* = 0.517).

### Baseline CONUT levels and DCR

**Figure 7:**
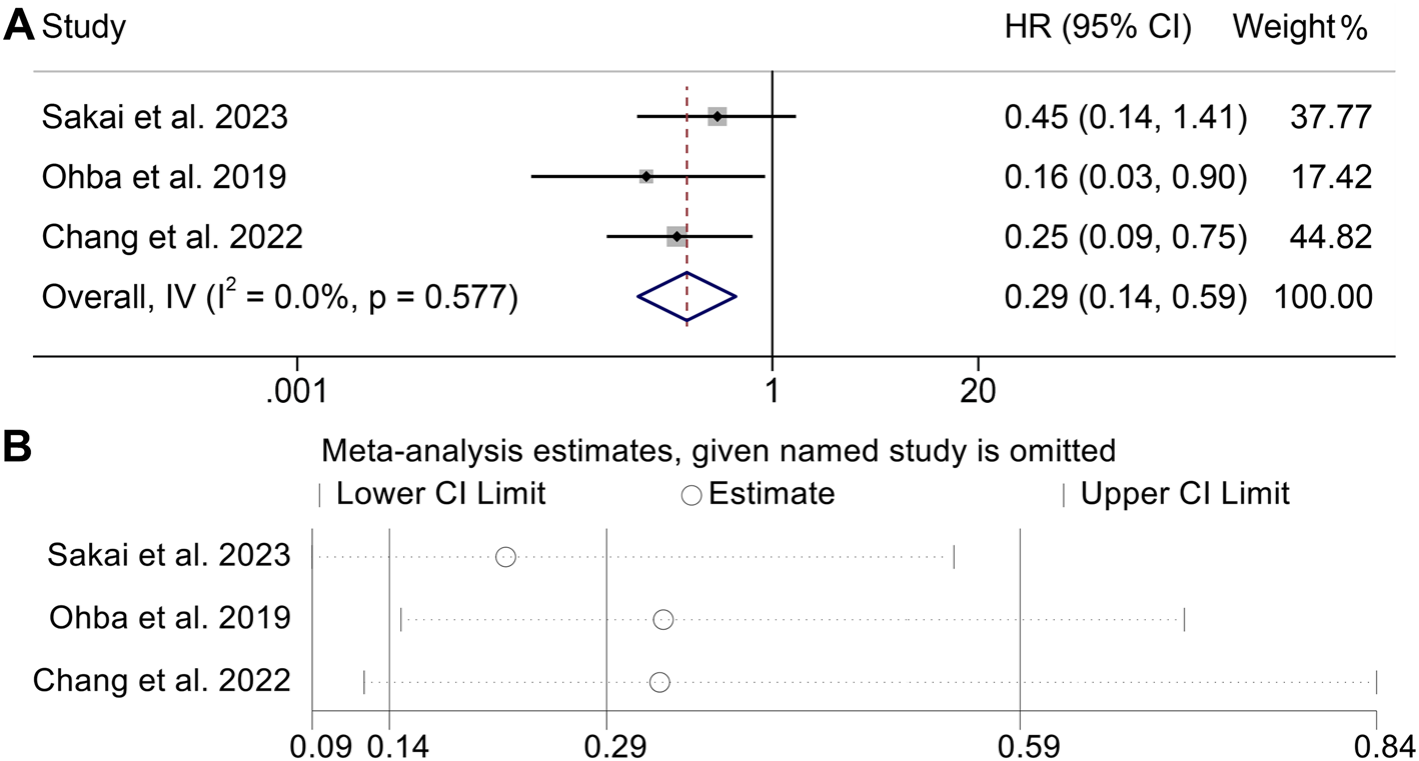
(**A**) Forest plots of the association between CONUT and disease control rate (DCR). *HR*, hazard ratio; *Cl*, confidence interval. (**B**) Sensitivity analysis of the association between CONUT and DCR.

Subsequently, we investigated the relationship between CONUT and DCR in cancer patients using data obtained from three independent studies encompassing 203 patients. Due to the heterogeneity was not significant (I2 = 0%, *p* = 0.577), the FEM was employed. According to Fig. 7A, the pooled HR was 0.29 (95% CI 0.14–0.59, *p* = 0.001). Based on the findings, it revealed that cancer patients who exhibit a high CONUT score had a decreased DCR when undergoing immunotherapy treatment. As Fig. 7B demonstrated, the overall predictive power of the DCR was unaffected by the absence of any one particular research.

## Discussion

The primary objective of our study was to investigate the prognostic value of the CONUT score in cancer patients undergoing ICI therapy. In this comprehensive meta-analysis of pertinent studies, a robust correlation has been established between a diminished CONUT score and a favorable OS and PFS, as well as an elevated ORR and DCR.

ICI has greatly increased the survival of tumor patients, but the overall clinically significant response rate is not satisfactory. Identifying patients who can distinguish between those who are likely to respond to immunotherapy will help increase patient benefit rates^36^. Clinical nutrition and immune-related indicator—CONUT is convenient and easy to obtain, which has been an excellent predictor in many ICI therapies. In our study, CONUT score can predict the effect of tumor immunotherapy, and tumor patients with lower CONUT scores will benefit more from immunotherapy. The prognostic implications of CONUT in cancer patients treated with ICIs can be elucidated through three fundamental components^37^. Specifically, albumin and total cholesterol are indicative of nutritional status, while lymphocytes serve as a reflection of immune functionality.

Clinically speaking, malnutrition frequent in cancer patients, and albumin is a widely used laboratory index in clinical practice to assess nutrition status^38^. At the same time, inflammation is inextricably linked to tumor progression, and there is also inflammation caused by immunotherapy. It is well known that the activation of the immune system after ICIs treatment is the major mechanism for the effectiveness of immunotherapy, and during ICI treatment, it may promote the occurrence of processes similar to inflammation^39,40^. Albumin is also one marker of the systemic inflammation in cancers^41^. The decrease in albumin levels is more pronounced in the middle and late stages of tumor patients, leading to hypoalbuminemia^42,43^. Hypoalbuminemia enhances the secretion of various inflammatory factors, such as interleukin-6 (IL-6) and tumor necrosis factor alpha (TNF-α), which further stimulates tumor inflammation progression^44,45^. Research has demonstrated that hypoalbuminemia is a risk factor for multiple types of tumors and also contributes to elevated mortality rates associated with tumors^46,47^. Cholesterol, an integral constituent of cellular membranes, exhibits a strong correlation with the proliferation of tumors, and cancer patients with low cholesterol levels often have a poor prognosis^48^. Cholesterol also regulates the innate and adaptive immune responses of a variety of immune cells in tumors^49^. Cholesterol in the tumor microenvironment can reduce T cell depletion and contribute to the attenuation of the immune response against cancers^50^. Studies have also shown that cholesterol can enhance the anti-tumor effects of natural killer cells in mice^51^. This partly explains why higher CONUT scores, which correspond to lower cholesterol levels, are less beneficial for cancer patients from ICI treatment. As we know, lymphocytes are of utmost importance in modulating the immune system's ability to combat cancer^52^. They effectively hinder the growth of tumor cells and exert suppressive effects on their invasive potential^53^. Those lymphocytes, especially tumor infiltrating lymphocytes (TILs), have been shown to actively contribute to the body's defense against tumor progression^54,55^. Fewer lymphocytes, corresponding to a higher CONUT score, tend to mean a worse immune status.

In this paper, a pioneering meta-analysis that aimed to validate the prognostic utility of the CONUT in predicting the therapeutic response of cancer patients undergoing ICI therapy was accomplished. Cancer patients with low CONUT scores may potentially experience a more favorable response to ICIs. The findings of this study can help cancer patients aid in developing effective treatment strategies that facilitate the administration of precise and reduce treatment costs for cancer patients.

Admittedly, there are still limitations to our research. First of all, the predominant proportion of studies encompassed within the meta-analysis consist of retrospective studies. Secondly, owing to constraints in the number of gathered research studies, the role of CONUT in post-ICI treatment could not be explored for individual tumors. Furthermore, our analysis was limited to studies published exclusively in English and focused on data from China and Japan. This geographical restriction should be taken into consideration when interpreting the findings. Further investigation and the inclusion of more extensive sample sizes are still required in order to validate the predictive significance of CONUT in the context of ICI treatment. First, we can conduct prospective studies to validate the predictive function of the CONUT score in relation to the impact of cancer immunotherapy. Second, An increased sample size is needed to analyze a specific type of cancer and discussed the predictive capability of the CONUT score for different tumor immunotherapies (“Supplementary information”).

## Conclusion

In conclusion, due to its well-established impact on the host's nutritional and immune status as well as cancer, the CONUT score could serve as a useful tool in predicting the therapeutic outcomes of ICIs in cancer patients.

### Supplementary Information

Supplementary Information.

## Data Availability

The data that support the findings of this study are available from the corresponding author upon reasonable request.
