# Supplementary material for: Prognostic value of controlling nutritional status on clinical and survival outcomes in cancer patients treated with immunotherapy
Source: Sci Rep. 2023 Oct 18;13:17715. doi: 10.1038/s41598-023-45096-1 (PMC10584918; doi:10.1038/s41598-023-45096-1)
Supplement: Supplementary file 1 — Supplementary Information. [file 41598_2023_45096_MOESM1_ESM.docx]

Supplementary Material

# Supplementary Data

# Supplementary material 1. The detailed search strategies for Pubmed

((((((Camrelizumab) OR (Sintilimab)) OR (Tislelizumab)) OR (Toripalimab)) OR (Envafolimab)) OR (((((((((((((((((((((((((((((((((((((((((((((((((anti-CTLA4 antibody) OR (anti-CTLA4 antibodies)) OR (Immune Checkpoint Inhibitors)) OR (Checkpoint Inhibitors, Immune)) OR (Immune Checkpoint Inhibitor)) OR (Checkpoint Inhibitor, Immune)) OR (Immune Checkpoint Blockers)) OR (Checkpoint Blockers, Immune)) OR (Immune Checkpoint Blockade)) OR (Checkpoint Blockade, Immune)) OR (Immune Checkpoint Inhibition)) OR (Checkpoint Inhibition, Immune)) OR (PD-L1 Inhibitors)) OR (PD L1 Inhibitors)) OR (PD-L1 Inhibitor)) OR (PD L1 Inhibitor)) OR (Programmed Death-Ligand 1 Inhibitors)) OR (Programmed Death Ligand 1 Inhibitors)) OR (PD-1-PD-L1 Blockade)) OR (Blockade, PD-1-PD-L1)) OR (PD 1 PD L1 Blockade)) OR (CTLA-4 Inhibitors)) OR (CTLA 4 Inhibitors)) OR (CTLA-4 Inhibitor)) OR (CTLA 4 Inhibitor)) OR (Cytotoxic T-Lymphocyte-Associated Protein 4 Inhibitors)) OR (Cytotoxic T Lymphocyte Associated Protein 4 Inhibitors)) OR (Cytotoxic T-Lymphocyte-Associated Protein 4 Inhibitor)) OR (Cytotoxic T Lymphocyte Associated Protein 4 Inhibitor)) OR (PD-1 Inhibitors)) OR (PD-1 Inhibitor)) OR (PD 1 Inhibitors)) OR (Inhibitor, PD-1)) OR (PD 1 Inhibitor)) OR (Programmed Cell Death Protein 1 Inhibitor)) OR (Programmed Cell Death Protein 1 Inhibitors)) OR (pembrolizumab)) OR (nivolumab)) OR (atezolizumab)) OR (ipilimumab)) OR (avelumab)) OR (tremelimumab)) OR (durvalumab)) OR (cemiplimab)) OR (anti-PD-1 antibodies)) OR (anti-PD-1 antibody)) OR (anti-PD-L1 antibody)) OR (anti-PD-L1 antibodies)) OR (Immune Checkpoint Inhibitors[MeSH Terms]))) AND (("Controlling Nutritional Status") OR (CONUT))

**The detailed search strategies for Google Scholar**

("Controlling Nutritional Status" and "Immune checkpoint inhibitors")

# Supplementary material 2. The calculation results.

OS

metan lnhr lnll lnul, label(namevar=study) eform fixed effect (HR) overall texts (130) xlabel (0.01, 1, 20

> 0) force boxsca(100)

Studies included: 7

Participants included: Unknown

Meta-analysis pooling of aggregate data

using the common-effect inverse-variance model

--------------------------------------------------------------------

Study | HR [95% Conf. Interval] % Weight

---------------------+----------------------------------------------

Zhao et al. 2023 | 3.607 1.686 7.717 10.09

Zhang et al. 2023 | 1.830 1.150 2.890 27.50

Sakai et al. 2023 | 1.942 1.104 3.417 18.29

Chen, L et al. 2022 | 1.310 0.730 2.350 17.08

Chang et al. 2022 | 2.056 1.031 4.098 12.26

Takemura et al. 2019 | 1.827 0.913 3.657 12.13

Ohba et al. 2019 | 4.000 1.020 20.000 2.64

---------------------+----------------------------------------------

Overall, IV | 1.937 1.521 2.467 100.00

--------------------------------------------------------------------

Test of overall effect = 1: z = 5.364 p = 0.000

Heterogeneity measures, calculated from the data

with Conf. Intervals based on non-central chi² (common-effect) distribution for Q

---------------------------------------------------------

Measure | Value df p-value

---------------------+-----------------------------------

Cochran's Q | 5.31 6 0.504

| -[95% Conf. Interval]-

H | 0.941 1.000 1.552

I² (%) | 0.0% 0.0% 58.5%

---------------------------------------------------------

H = relative excess in Cochran's Q over its degrees-of-freedom

I² = proportion of total variation in effect estimate due to between-study heterogeneity (based on Q)

Note: with metan version 4 and above, the preferred syntax is for xlabel()

to contain a standard Stata numlist, so e.g. xlabel(.01 1 200); see help numlist

. graph export "C:\Users\lilong\Desktop\待研究论文\CONUT与免疫治疗应答\计算结果\OS 森林图.pdf", as(pdf) repl

> ace

(file C:\Users\lilong\Desktop\待研究论文\CONUT与免疫治疗应答\计算结果\OS 森林图.pdf written in PDF format)

.

. metabias lnhr selnhr,egger graph

Note: default data input format (theta, se_theta) assumed.

Tests for Publication Bias

Begg's Test

adj. Kendall's Score (P-Q) = 11

Std. Dev. of Score = 6.66

Number of Studies = 7

z = 1.65

Pr > |z| = 0.099

z = 1.50 (continuity corrected)

Pr > |z| = 0.133 (continuity corrected)

Egger's test

------------------------------------------------------------------------------

Std_Eff | Coef. Std. Err. t P>|t| [95% Conf. Interval]

-------------+----------------------------------------------------------------

slope | .0585638 .3680657 0.16 0.880 -.8875792 1.004707

bias | 1.921791 1.128487 1.70 0.149 -.9790779 4.82266

------------------------------------------------------------------------------

metaninf lnhr selnhr, label(namevar=study) fixed eform

------------------------------------------------------------------------------

Study omitted | Estimate [95% Conf. Interval]

-------------------+----------------------------------------------------------

Zhao et al. 2023 | 1.8066511 1.4002572 2.330992

Zhang et al. 2023 | 1.9795328 1.4904742 2.6290629

Sakai et al. 2023 | 1.9361666 1.4820242 2.5294738

Chen, L et al. 2022| 2.0998676 1.6104757 2.7379763

Chang et al. 2022 | 1.9211884 1.4843748 2.4865448

Takemura et al. 2019| 1.9529591 1.5092224 2.5271618

Ohba et al. 2019 | 1.8995643 1.4869952 2.4266012

-------------------+----------------------------------------------------------

Combined | 1.9372325 1.5214187 2.4666909

------------------------------------------------------------------------------

# PFS

metan lnhr lnll lnul, label(namevar=study) eform random effect (HR) overall texts (130) xlabel (0.01, 1, 2

> 00) force boxsca(100)

Studies included: 8

Participants included: Unknown

Meta-analysis pooling of aggregate data

using the random-effects inverse-variance model

with DerSimonian-Laird estimate of tau²

--------------------------------------------------------------------

Study | HR [95% Conf. Interval] % Weight

---------------------+----------------------------------------------

Zhao et al. 2023 | 2.355 1.012 5.481 12.61

Zhang et al. 2023 | 2.343 1.297 4.233 17.64

Sakai et al. 2023 | 1.848 1.131 3.019 20.02

Chen, X et al. 2022 | 3.862 1.186 12.577 8.23

Chen, L et al. 2022 | 1.080 0.520 1.850 16.67

Chang et al. 2022 | 1.299 0.521 3.239 11.52

Takemura et al. 2019 | 12.253 1.585 94.734 3.39

Ohba et al. 2019 | 5.882 2.128 16.667 9.92

---------------------+----------------------------------------------

Overall, DL | 2.217 1.484 3.311 100.00

--------------------------------------------------------------------

Test of overall effect = 1: z = 3.889 p = 0.000

Heterogeneity measures, calculated from the data

with Conf. Intervals based on Gamma (random-effects) distribution for Q

---------------------------------------------------------

Measure | Value df p-value

---------------------+-----------------------------------

Cochran's Q | 13.41 7 0.063

| -[95% Conf. Interval]-

H | 1.384 1.000 2.127

I² (%) | 47.8% 0.0% 77.9%

---------------------------------------------------------

H = relative excess in Cochran's Q over its degrees-of-freedom

I² = proportion of total variation in effect estimate due to between-study heterogeneity (based on Q)

Heterogeneity variance estimates

-----------------------------------

Method | tau²

---------------------+-------------

DL | 0.1466

-----------------------------------

Note: with metan version 4 and above, the preferred syntax is for xlabel()

to contain a standard Stata numlist, so e.g. xlabel(.01 1 200); see help numlist

. graph export "C:\Users\lilong\Desktop\待研究论文\CONUT与免疫治疗应答\计算结果\PFS 森林图.pdf", as(pdf) rep

> lace

(file C:\Users\lilong\Desktop\待研究论文\CONUT与免疫治疗应答\计算结果\PFS 森林图.pdf written in PDF format)

.

Note: default data input format (theta, se_theta) assumed.

Tests for Publication Bias

Begg's Test

adj. Kendall's Score (P-Q) = 12

Std. Dev. of Score = 8.08

Number of Studies = 8

z = 1.48

Pr > |z| = 0.138

z = 1.36 (continuity corrected)

Pr > |z| = 0.174 (continuity corrected)

Egger's test

------------------------------------------------------------------------------

Std_Eff | Coef. Std. Err. t P>|t| [95% Conf. Interval]

-------------+----------------------------------------------------------------

slope | -.1204362 .4451357 -0.27 0.796 -1.209644 .9687717

bias | 2.301696 1.153837 1.99 0.093 -.5216415 5.125033

------------------------------------------------------------------------------

# ORR

metan lnhr lnll lnul, label(namevar=study) eform fixed effect (OR) overall texts (130) xlabel (0.001, 1, 2

> 0) force boxsca(100)

Studies included: 4

Participants included: Unknown

Meta-analysis pooling of aggregate data

using the common-effect inverse-variance model

--------------------------------------------------------------------

Study | OR [95% Conf. Interval] % Weight

---------------------+----------------------------------------------

Zhao et al. 2023 | 0.229 0.055 0.957 17.94

Sakai et al. 2023 | 0.780 0.260 2.340 30.32

Chang et al. 2022 | 0.441 0.164 1.189 37.30

Ohba et al. 2019 | 0.429 0.087 2.101 14.44

---------------------+----------------------------------------------

Overall, IV | 0.464 0.254 0.850 100.00

--------------------------------------------------------------------

Test of overall effect = 1: z = -2.486 p = 0.013

Heterogeneity measures, calculated from the data

with Conf. Intervals based on non-central chi² (common-effect) distribution for Q

---------------------------------------------------------

Measure | Value df p-value

---------------------+-----------------------------------

Cochran's Q | 1.82 3 0.611

| -[95% Conf. Interval]-

H | 0.778 1.000 1.765

I² (%) | 0.0% 0.0% 67.9%

---------------------------------------------------------

H = relative excess in Cochran's Q over its degrees-of-freedom

I² = proportion of total variation in effect estimate due to between-study heterogeneity (based on Q)

Note: with metan version 4 and above, the preferred syntax is for xlabel()

to contain a standard Stata numlist, so e.g. xlabel(.001 1 20); see help numlist

. graph export "C:\Users\lilong\Desktop\待研究论文\CONUT与免疫治疗应答\计算结果\ORR 森林图.pdf", as(pdf) rep

> lace

(file C:\Users\lilong\Desktop\待研究论文\CONUT与免疫治疗应答\计算结果\ORR 森林图.pdf written in PDF format)

. metabias lnhr selnhr,egger graph

Note: default data input format (theta, se_theta) assumed.

Tests for Publication Bias

Begg's Test

adj. Kendall's Score (P-Q) = 0

Std. Dev. of Score = 2.94

Number of Studies = 4

z = 0.00

Pr > |z| = 1.000

z = -0.34 (continuity corrected)

Pr > |z| = 1.000 (continuity corrected)

Egger's test

------------------------------------------------------------------------------

Std_Eff | Coef. Std. Err. t P>|t| [95% Conf. Interval]

-------------+----------------------------------------------------------------

slope | .2914609 1.381442 0.21 0.852 -5.652404 6.235326

bias | -1.745884 2.237905 -0.78 0.517 -11.37481 7.883043

metaninf lnhr selnhr, label(namevar=study) fixed eform

------------------------------------------------------------------------------

Study omitted | Estimate [95% Conf. Interval]

-------------------+----------------------------------------------------------

Zhao et al. 2023 | .54180127 .27785566 1.0564787

Sakai et al. 2023 | .37040615 .17945053 .7645601

Chang et al. 2022 | .47864166 .22295626 1.0275462

Ohba et al. 2019 | .47046408 .24462761 .90478939

-------------------+----------------------------------------------------------

Combined | .46423907 .25352541 .85008407

------------------------------------------------------------------------------

# .DCR

. metan lnhr lnll lnul, label(namevar=study) eform fixed effect (HR) overall texts (130) xlabel (0.001, 1, 2

> 0) force boxsca(100)

Studies included: 3

Participants included: Unknown

Meta-analysis pooling of aggregate data

using the common-effect inverse-variance model

--------------------------------------------------------------------

Study | HR [95% Conf. Interval] % Weight

---------------------+----------------------------------------------

Sakai et al. 2023 | 0.450 0.140 1.410 37.77

Ohba et al. 2019 | 0.160 0.030 0.900 17.42

Chang et al. 2022 | 0.250 0.090 0.750 44.82

---------------------+----------------------------------------------

Overall, IV | 0.289 0.142 0.587 100.00

--------------------------------------------------------------------

Test of overall effect = 1: z = -3.430 p = 0.001

Heterogeneity measures, calculated from the data

with Conf. Intervals based on non-central chi² (common-effect) distribution for Q

---------------------------------------------------------

Measure | Value df p-value

---------------------+-----------------------------------

Cochran's Q | 1.10 2 0.577

| -[95% Conf. Interval]-

H | 0.742 1.000 1.921

I² (%) | 0.0% 0.0% 72.9%

---------------------------------------------------------

H = relative excess in Cochran's Q over its degrees-of-freedom

I² = proportion of total variation in effect estimate due to between-study heterogeneity (based on Q)

Note: with metan version 4 and above, the preferred syntax is for xlabel()

to contain a standard Stata numlist, so e.g. xlabel(.001 1 20); see help numlist

. graph export "C:\Users\lilong\Desktop\待研究论文\CONUT与免疫治疗应答\计算结果\DCR 森林图.pdf", as(pdf) rep

> lace

(file C:\Users\lilong\Desktop\待研究论文\CONUT与免疫治疗应答\计算结果\DCR 森林图.pdf written in PDF format)

metaninf lnhr selnhr, label(namevar=study) fixed eform

------------------------------------------------------------------------------

Study omitted | Estimate [95% Conf. Interval]

-------------------+----------------------------------------------------------

Sakai et al. 2023 | .22064711 .08974208 .54250073

Ohba et al. 2019 | .32709953 .14980133 .71424007

Chang et al. 2022 | .32469389 .12490045 .84408116

-------------------+----------------------------------------------------------

Combined | .28879631 .14202811 .58723101

------------------------------------------------------------------------------

.

.
